# Bipolar Disorder

**DOI:** 10.1155/2012/525837

**Published:** 2012-04-04

**Authors:** Cristina Colombo, Andrea Fossati, Francesc Colom

**Affiliations:** ^1^Neuropsychiatric Science Department, San Raffaele Hospital, School of Medicine, Universita' Vita Salute San Raffaele, 20127 Milano, Italy; ^2^Neuropsychiatric Sciences Department, San Raffaele Hospital, Faculty of Psychology, Università Vita Salute San Raffaele, 20127 Milano, Italy; ^3^Psychoeducation and Psychological Treatments Area, Barcelona Bipolar Disorders Unit, Bipolar Disorders Program, IDIBAPS-CIBERSAM, Hospital Clinic Barcelona, 08038 Barcelona, Spain

According to the World Health Organization, bipolar disorder is the 6th leading cause of disability in the world and it affects about 5% of the population with dangerous repercussions on multiple aspects in a person's life. The depressive phase of bipolar disorder is often very severe, and suicide is a major risk factor. Studies on the causes of the disorder focus on environmental triggers such as unexpected stressors and circadian variations and on genetic contributions.

Moreover, psychobiological factors seem to play an outstanding role not only in the aetiology of the disorder but also in its outcome and, potentially, in the response or lack of response to pharmacological and psychological interventions. Newer staging models suggest elevated levels of several cytokines and BDNF decreased levels for poor responders. Papers in this special issue treat a vast variety of topics regarding bipolar disorder, reflecting the complexity and the multifactorial aetiology of the disorder. Starting from point of view which considers the intricacy of the disorder, the review “Functional outcome in bipolar disorder: the big picture” gives us a thorough overview on the psychosocial implications after the onset of the illness which influence the quality of the patient's life on a global level. It considers options to enhance patient care and to provide an overall improvement in the functional outcome with therapies such as interpersonal and social rhythms therapy which has been shown to be effective in the long-term clinical management of the disorder.

When we consider the predisposition of the general population toward mood disorders it is important to take into account the detection of depressive and manic cognitive vulnerability in healthy subjects. F. Raes et al. used self-administered rating scales which address the tendency to react negatively or positively to events based on the person's “bipolar tendency”. Interestingly, they find that in subjects with a subclinical bipolar tendency, their tendency correlated significantly with a self-perception of failure in negative events and a self perception of success in positive events suggesting that these mirrored cognitive features both form part of vulnerability to bipolar disorder. Another interesting aspect is the early detection of bipolar disorder, in fact R. Jairam's review examines the controversies tied to the onset and diagnosis of bipolar disorder in children and adolescents. Authors illustrate the difficulty in distinguishing severe mood dysregulation from bipolar disorder highlighting the lack of an effective treatment for this spectrum of childhood disorders.

Another issue involved in the difficulty of an unambiguous diagnosis of bipolar disorder is substance abuse: in fact it is difficult to determine whether substance abuse occurs during an episode itself, or if an abuse leads to the onset of an illness episode. S. Theodore's paper clearly discusses the role of substance abuse in bipolar disorder and the difficulties of avoiding misdiagnosis and of determining the presence of comorbidities. They find that discrepancies in clinicians are common as symptoms can often overlap and suggest the use of symptom time lines to better distinguish and document their origin. In recent years, numerous researches have not focused only on concerns regarding the disorder's symptomatology and comorbidity, but there has been a growing interest in studying the link between chronobiology and the pathogenesis of mood disturbance. The paper “Seasonality and sleep: a clinical study on euthymic mood disorder patients” discusses the impact of “rhythmical” factors such as seasonality, sleep, and chronotype (chronobiological factors that can strongly influence the course of mood disorders) with the aim of improving our knowledge of somatic treatment strategies.

Lastly, new implications in the psychological treatment of bipolar disorder are discussed in W. Marchand work. This paper highlights critical features of bipolar disorder including anxiety and on a level of more severe aspects, an elevated suicidal risk. Various studies argue that cortical midline structures are involved in the emotional dysregulation in mood disorders. Authors propose that mindfulness-based interventions, which according to neuroimaging studies modulate cortical midline structures, may improve cognitive and emotional dysfunctions typically observed in patients with affective disorders.

In this special issue, we have brought together multiple disciplines and multiple nationalities, treating various aspects of this intricate illness hoping that these new hypotheses, ideas, and findings will stimulate future research to elucidate aspects of the disorder aetiology, treatment and improve patients' quality of life.

*Cristina Colombo* *Cristina Colombo* *Andrea Fossati* *Andrea Fossati* *Francesc Colom* *Francesc Colom*

